# Perspectives and Expertise in Establishing a Therapeutic Drug Monitoring Programme for Challenging Childhood Cancer Patient Populations

**DOI:** 10.3389/fonc.2021.815040

**Published:** 2022-01-06

**Authors:** Shelby Barnett, Victoria Holden, Quentin Campbell-Hewson, Gareth J. Veal

**Affiliations:** ^1^ Newcastle University Centre for Cancer, Newcastle University, Newcastle upon Tyne, United Kingdom; ^2^ Leeds General Infirmary, Leeds, United Kingdom; ^3^ Great North Children’s Hospital, Newcastle upon Tyne, United Kingdom

**Keywords:** Therapeutic drug monitoring, children, cancer, chemotherapy, dosing adaptation

## Abstract

The utility of Therapeutic Drug Monitoring (TDM) in the setting of childhood cancer is a largely underused tool, despite the common use of cytotoxic chemotherapeutics. While it is encouraging that modern advances in chemotherapy have transformed outcomes for children diagnosed with cancer, this has come at the cost of an elevated risk of life-changing long-term morbidity and late effects. This concern can limit the intensity at which these drugs are used. Widely used chemotherapeutics exhibit marked inter-patient variability in drug exposures following standard dosing, with fine margins between exposures resulting in toxicity and those resulting in potentially suboptimal efficacy, thereby fulfilling criteria widely accepted as fundamental for TDM approaches. Over the past decade in the UK, the paediatric oncology community has increasingly embraced the potential benefits of utilising TDM for particularly challenging patient groups, including infants, anephric patients and those receiving high dose chemotherapy. This has been driven by a desire from paediatric oncologists to have access to clinical pharmacology information to support dosing decisions being made. This provides the potential to modify doses between treatment cycles based on a comprehensive set of clinical information, with individual patient drug exposures being used alongside clinical response and tolerability data to inform dosing for subsequent cycles. The current article provides an overview of recent experiences of conducting TDM in a childhood cancer setting, from the perspectives of the clinicians, scientists and pharmacists implementing TDM-based dosing recommendations. The ongoing programme of work has facilitated investigations into the validity of current approaches to dosing for some of the most challenging childhood cancer patient groups, with TDM approaches now being expanded from well-established cytotoxic drugs through to newer targeted treatments.

## Introduction

Every year in the UK, there are approximately 1,800 children diagnosed with cancer, with incidence rates highest in children less than 5 years of age (1). Only a small proportion of the most common childhood cancers are curable with local therapy and prior to the widespread adoption of systemic cytotoxic chemotherapy, cure was rare. The use of increasingly complex chemotherapy regimes has been transformative for children and young people affected by malignancy, with 5 year survival rates for children with cancer in the UK rising from 44% between 1973-77 to 84% between 2011-15 (2). Increased survival has been strongly associated with increased intensity of cytotoxic therapy, an approach clearly demonstrated for cancers such as neuroblastoma (3) and Ewing sarcoma (4).

While it is encouraging that modern advances in cancer treatment now mean that over 70% of childhood cancer patients will survive for twenty years or more following diagnosis, this comes at the cost of an elevated risk of life-changing long-term morbidity and late effects (5, 6). With an estimated 40,000+ childhood cancer survivors now living in the UK, this is clearly a major issue, both in terms of the quality of life experienced by those affected, as well as the financial impact on both the individual and the NHS (7, 8). On the other hand, undertreatment to avoid toxicity risks compromising survival.

The vast majority of childhood cancer patients are treated with non-selective cytotoxic anticancer drugs, with significant potential to damage host tissue at doses used to achieve anti-tumour activity. Widely used drugs which are effective against a wide range of childhood cancers include carboplatin and cisplatin, cyclophosphamide, vincristine, doxorubicin and etoposide, and have been a mainstay of treatment for several decades. However, these drugs are associated with a plethora of toxicities and late effects including organ dysfunction, hearing loss, infertility, secondary malignancies and cognitive problems (9–13). As drug toxicity is dependent on immediate and cumulative dose for the majority of chemotherapeutic drugs, drug exposure is clearly an important factor.

The utility of therapeutic drug monitoring (TDM) is widely used across a range of disease specialties and drug classes, including antibiotics, antipsychotics, anticonvulsants and immunosuppressants (14). However, it has remained an underused tool in an oncology setting, despite a number of published studies highlighting its potential clinical benefit (15–18). More recently, studies supportive of TDM approaches for newer targeted anticancer drugs have been published (19–22). The understated use of TDM for well established cytotoxic drugs is particularly surprising, when we consider that these drugs commonly exhibit the characteristics widely accepted as fundamentals for utilising TDM approaches. These include marked inter-patient variability in drug exposures following standard dosing, the existence of a narrow therapeutic window, with fine margins between exposures resulting in toxicity and those resulting in potentially suboptimal efficacy, and evidence for relationships between drug exposure and clinical endpoints (23). A recent review on the use of TDM for the widely used anticancer drug carboplatin, provides a good level of detail on this how this drug meets the characteristics commonly associated with TDM (18).

While there are certainly challenges in implementing TDM in an oncology setting, including the use of traditional dosing regimens and common use of drug combinations, as highlighted in some excellent reviews on the subject (24–26), these hurdles are certainly not unsurmountable if the problem is approached in the right way. Indeed, TDM approaches have been shown to be beneficial and are commonly used for the anticancer drugs methotrexate and busulfan across a range of cancer types. Over the past decade in the UK, the paediatric oncology community has increasingly embraced the potential benefits of utilising TDM approaches for particularly challenging patient groups, including neonates and infants, anephric patients and those receiving high dose chemotherapy regimens. This has very much been led by a desire from paediatric oncologists to have access to clinical pharmacology information as an additional tool when making difficult dosing decisions. This approach means that a clinician can modify doses between cycles of treatment based on a more comprehensive set of clinical information, with individual patient drug exposure following the initial drug dose being used alongside clinical response and tolerability data to inform dosing for subsequent cycles. The current article looks at the recent experiences of conducting TDM in a childhood cancer setting from the perspectives of the clinicians requesting the use of TDM for their patients, the scientists carrying out sample clinical sample analysis, and the pharmacists implementing TDM-based dosing recommendations.

## View From the Paediatric Oncologist

Delivery of the optimum dose of chemotherapy is crucial if we are to achieve the best survival outcome at the least toxic cost to our patients. Drug exposure is known to be closely related to a range of factors including body mass and composition and drug elimination and detoxification, usually by the renal or hepatic systems (27, 28). Standard dosing of treatment may be assumed for children who lie within the normal range of these parameters, but a meaningful proportion of children lie outside them (29, 30). For patient size, most concern has been with small or very young children and infants, who it has been feared might be overdosed with standard dosing regimens. However, larger children or those with disproportionate body fat may be as problematic, particularly where chemotherapy dosing is capped. Children with immature and developing liver and kidney function, as well as those with diminished function following disease, physical injury or drug toxicity, might be overexposed to drugs. For the large part, the availability of dosing guidelines that we can have confidence in has been unachievable in these patient groups, partly due to the relatively small numbers of cases that we are presented with.

Concerns about body size have led to reticence and anxiety surrounding the use of these highly effective chemotherapy drugs and also guidelines on “safe” dosing. These typically set a cut-off weight and commonly adopt a weight-based dose calculation, as opposed to the standard surface area-based dosing approach usually employed. Weight-based dosing typically yields a lower drug exposure than dosing based on surface area, although the implication is that it is equivalent. In the previous European Paediatric Soft Tissue Sarcoma RMS 2005 Rhabdomyosarcoma study, a child of 10 kg would receive 30-40% less vincristine, ifosfamide, actinomycin and doxorubicin, when calculated by weight as opposed to surface area (31). Similarly, for the previous SIOPEN High Risk Neuroblastoma protocol, a child of 12kg similarly would receive 30-40% lower doses of carboplatin, vincristine, etoposide, cisplatin and cyclophosphamide (32). Unless these dose reductions are justified by a difference in the handling of these drugs in smaller children, then these patients may be receiving a substantial under-treatment, which may have fatal consequences. In this respect, a review looking at currently available evidence for dosing guidance of a wide range of anticancer drugs used in infants and neonates has recently been published and provides a valuable tool (33).

Altered drug elimination by temporary or permanent renal or liver dysfunction appears even more unpredictable than size. Most drugs are cleared by more than one mechanism, making it very challenging to establish reliable guidelines. In this scenario, current guidelines are even more crude than size guidance, with dosing being reduced by 50% or even involving the omission of drugs altogether (34). In children out-with the usual norms of size and excretion, the clinician faces the anxiety provoking choice of accepting the recommended dose reductions or administering doses with the potential to achieve maximum efficacy. In the latter case, this would be undertaken in the knowledge that severe toxicity would leave them open to the charge of negligently overdosing, with a lack of guideline or evidence support for the decisions taken.

In our experience, utilising a TDM approach in these challenging patient groups provides evidence to support the administration of chemotherapy dosing regimens most likely to achieve the best outcomes. In children who would have what amount to dose reductions due to their size, it has allowed us to tailor doses to the actual patient. This has almost always resulted in dosing regimens more equivalent to those used to dose older children, thus calling into question the widely used lower weight-based dosing guidelines. In most cases TDM enables us to give higher doses of treatment, with the expectation of better response rates and survival. The ability to therapeutically monitor repeated cycles of a wide range of drugs has repeatedly allowed us to adapt treatment for very young infants as they progress through organ maturation, without compromising treatment.

## View From the Scientist

**Figure 1:**
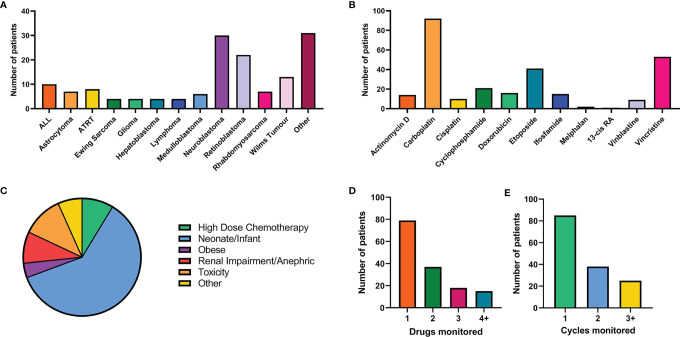
Summary of patient recruitment for the first 150 patient recruited on to the NCCPG TDM 2018 study. **(A)** Breakdown of tumour types and number of patients (cut off level for each tumour type of 3 patients). **(B)** Number of patients receiving each chemotherapeutic agent of interest. **(C)** Patient recruitment by hard-to-treat group. **(D)** The number of drugs monitored for each patient. **(E)** Number of cycles of chemotherapy monitored using TDM for each patient.

A formal clinical trial to allow the collection and analysis of patient information alongside the quantification of drug levels in defined groups of childhood cancer patients was initiated in 2019 (ISRCTN 10139334). This study was established due to an increasing number of clinical requests to monitor hard-to-treat childhood cancer patients, where drug exposure may be altered relative to older children. This formal clinical study has allowed us to collect patient clinical information relating to toxicity/efficacy alongside pharmacokinetic data, in order to better assess dosing regimens and understand relationships between drug exposure and clinical outcome in these challenging groups. The study opened for recruitment in April 2019, since then over 150 patients (average 5 patients per month) have been recruited from 16 primary treatment centres across the UK. Focusing on the first 150 patients recruited between April 2019 and October 2021 (**Figure 1**), a range of tumour types, hard-to-treat groups and chemotherapy regimens have been enrolled onto the study. The highest recruiting tumour types are neuroblastoma and retinoblastoma (**Figure 1A**), likely due to the established practice of carboplatin TDM (**Figure 1B**) (18). Additional tumour types in the ‘other’ grouping in this figure include infantile myofibromatosis, ependymoma, kidney tumours, Inflammatory myofibroblastic tumour and metastatic yolk sac tumour. Carboplatin is the drug most commonly analysed, with TDM carried out for over 60% of patients recruited onto the study, followed by vincristine (35%) and etoposide (28%), as these three drugs are commonly given in combination. Neonates and infants represent the highest recruiting group of the study to date, accounting for nearly two thirds of patients (**Figure 1C**). The second highest recruiting group included patients where there were concerns regarding poor tolerability to initial dosing regimens (11%). For these ‘toxicity’ patients, TDM is used to determine if the standard dose is contributing to excessive exposure and subsequent toxicities. Alternatively, TDM can be used to determine if suitable exposures are being achieved in patients who have experienced excessive toxicity and are receiving dosage reductions. The remaining hard-to-treat groups (high dose chemotherapy, obesity, renal impairment and other) had an equal spread of numbers between them, accounting for 4-9% of the patients for each group (**Figure 1C**). Patients recruited under the ‘other’ category included patients with low body weight for age, rare genetic conditions and hepatic dysfunction. It is important to note that patients may fall within multiple hard-to-treat groups, but are represented here as their primary group.

**Figure 2:**
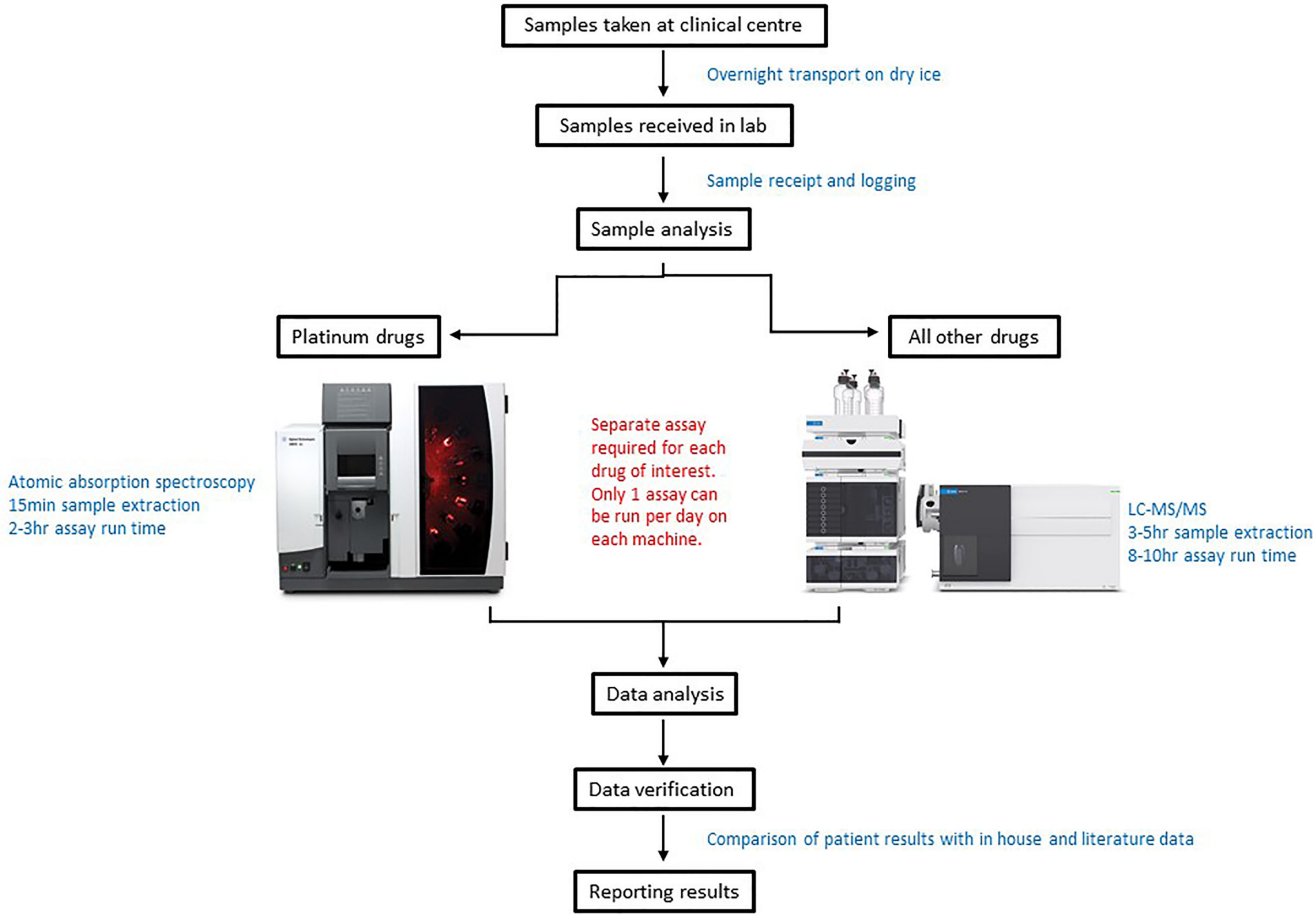
Schematic diagram of the sample analysis processes for patients utilising a Therapeutic Drug monitoring (TDM) approach to treatment.

As patients often receive multiple chemotherapeutic agents as part of their treatment, in many cases TDM was conducted for more than one drug per patient (**Figure 1E**). From a laboratory perspective co-ordination of patient sample analysis on such a large scale can be challenging (**Figure 2**). Of the 150 patients on the study 40% received TDM on more than one occasion (**Figure 1E**), with two patients being monitored on as many as eight TDM cycles. Whilst challenging however, this can provide valuable information on intra-patient variability for a particular drug, which can be a key factor influencing the likely success of the TDM approach to treatment. In addition, just under half (47%) of the patients on the study were monitored for more than one drug (**Figure 1D**). Carboplatin is the only drug where TDM is conducted in real time, i.e. samples are received, analysed and the results reported on day 2 of treatment in order to adjust the dose on day 3 (18). For the remaining drugs (**Figure 1B**), the results are reported ahead of the next cycle of chemotherapy, in order to make informed dose adjustments as required. This is partly a result of the more complex sample extraction and analysis used for these drugs compared to platinum containing agents (**Figure 2**). Furthermore, if more than one chemotherapeutic agent is being monitored then a separate assay has to be conducted for each drug of interest. Consequently, it may take several days to complete the analysis for a single patient. This is something that ideally will be simplified in the future, with the development of validated multi-drug assays to quantifying levels of several anticancer drugs simultaneously. Co-ordination of patient sample shipment, analysis and results, has been an important aspect of this complex multi-centre TDM study, to ensure that results are reported in a timely manner for all patients. This can require batching patient samples to reduce the number of assays and prioritising experiments based on when patient results are needed for clinical care.

## View From the Pharmacist

A significant challenge for a children’s cancer pharmacist when prescribing or verifying chemotherapy prescriptions, is the choice of chemotherapy doses in certain patient groups. These include infants, pre-term babies and children at extremes of body mass index (BMI) for age, as well as children with renal or hepatic impairment. In addition, the pharmacist is often asked for advice on chemotherapy dosing for children who have developed toxicity following previous courses of treatment.

The challenge of dosing in infants is compounded by a lack of consistent guidance in national and international treatment guidelines and clinical trials, with artificial cut-off points for mg/kg dosing in infants leading to sometimes large discrepancies in dose as compared to patients receiving mg/m^2^ dosing. Similarly, despite recent ASCO guidance in adults advising that dosing in obesity should be based on actual body weight (35), there is no national position statement or standard advice for dosing chemotherapy in obesity in children. In a paediatric setting, decisions on dose capping are often decided on an individual patient basis considering the patient’s BMI, renal and hepatic function, the drug’s toxicity profile and most importantly the clinician and pharmacist’s previous experiences with the drug in a similar patient group. Choice of dose in children with renal and liver impairment can be difficult due to varying or lack of advice in treatment guidelines, and minimal or cautious advice from the drug companies. There is also a lack of consistent dose modification guidance in protocols for patients who have experienced adverse effects on previous courses of treatment. The pharmacist is left juggling a delicate balance between desired therapeutic outcome and acceptable toxicity in such patients.

Carboplatin represents the drug most commonly administered using a TDM approach in childhood cancer in the UK, ensuring safe dosing in infants, children with renal impairment and patients receiving high dose chemotherapy prior to stem cell rescue. This is based on evidence from studies showing clear relationships between carboplatin drug exposure (AUC) and clinical outcome (18). The carboplatin dose is usually fractionated over 3 days and the AUC from day one used to advice the dose on day 3 (18). Whilst this is invaluable in terms of delivering an accurate dose of carboplatin, it can provide practical challenges for the children’s cancer pharmacist, as the carboplatin dose needs to be amended on the electronic prescribing system. Instead of prescribing the full carboplatin dose on day 1, the prescription is amended so that a third of the proposed dose is prescribed on each the first two days of treatment and then the third day left blank until the AUC results are known. The pharmacist liaises with both pharmacy aseptics and the research nurse team, to ensure the drug is available to start treatment early in the morning, and the research team have the staff resource to take the appropriately timed blood samples and to arrange transport of the samples for analysis. Once information on drug exposure is available, in terms of the observed AUC on day 1, the pharmacist and clinician review the data together, to agree a dose to prescribe for the third treatment day. The original prescription is then amended and a new dose prepared, often at short notice, by the aseptics unit. Whilst this is achievable for hospitals with an onsite aseptics unit, such an approach may not be possible for centres that outsource their chemotherapy. Experience has shown that communication and team working with other members of the multi-disciplinary team (MDT) is key to the successful delivery of real time TDM.

Based on positive experiences with carboplatin dosing, expansion of TDM approaches to a wide range of commonly used drugs now provides invaluable information to aid pharmacists with chemotherapy dosing decisions on a regular basis. It is reassuring to know that chemotherapy drug levels can be determined in individual patients, and the results provided can help guide dosing for subsequent courses of treatment. Suitable patients for TDM are identified by the pharmacists or clinicians at MDT meetings. After analysis of TDM samples, the pharmacist is provided with information that shows the drug level achieved, as compared to the usual therapeutic range. The results are used to determine if the dosage can remain the same or should be reduced or increased for the next chemotherapy cycle. As well as recommending TDM for the previously highlighted patient groups, the pharmacist may recommend TDM when a child has had significant adverse effects with a drug. Establishing whether or not toxicity is potentially related to excessive drug levels can help guide future patient management. For example, if vincristine dose is reduced by 50% due to drug-induced neuropathy, it is important to know that a potentially efficacious drug exposure is still being achieved at the reduced dose level, allowing this dose to be maintained for the remainder of treatment to minimise further neurological toxicity.

## Discussion

The successful utility of TDM dosing for patients treated across UK paediatric oncology primary treatment centres requires a collective commitment and effective teamwork between the scientists, research nurses, clinicians and pharmacists involved. The ongoing programme of work has facilitated investigations into the validity of current approaches to dosing for some of the most challenging childhood cancer patient groups. As an example of the impact of the information being generated from this study, we recently reported on current approaches to vincristine dosing in infants and neonates relative to older children (36). The results showed the feasibility of utilising a TDM treatment approach in this patient group and importantly, highlighted that infants receiving vincristine doses <0.05mg/kg were achieving significantly lower exposures compared to those dosed at ≥0.05mg/kg, and older children dosed at 1.5 mg/m^2^. Furthermore, infants with lower exposures tolerated dose increases well, suggesting that infants should not be initiating treatment with some of the lower mg/kg dosing regimens currently being used. It is hoped that similar analyses will be feasible for additional drugs being monitored on the study, leading to the generation of further data to support future dosing in these challenging patient populations. This approach to treatment is now being expanded from well established cytotoxic drugs through to newer targeted treatments, as they become increasingly utilised in a childhood cancer setting.

## Author Contributions

The manuscript was conceptualized by GV. All authors contributed to the writing of the article and approved the submitted version.

## Funding

The TDM programme of work is currently supported by the Little Princess Trust (CCLGA 2021 08 Veal), Cancer Research UK (C9380/A25138) and the Experimental Cancer Medicine Centre Network (C9380/A25169), with previous funding from the National Institute for Health Research (NIHR) Research for Patient Benefit programme (PB-PG-1216-20032).

## Author Disclaimer

The views expressed are those of the authors and not necessarily those of the funders, the NIHR or the Department of Health and Social Care. None of the funding bodies played a role in the study design, the collection, analysis or interpretation of data, the writing of the report or the decision to submit the article for publication.

## Conflict of Interest

The authors declare that the research was conducted in the absence of any commercial or financial relationships that could be construed as a potential conflict of interest.

## Publisher’s Note

All claims expressed in this article are solely those of the authors and do not necessarily represent those of their affiliated organizations, or those of the publisher, the editors and the reviewers. Any product that may be evaluated in this article, or claim that may be made by its manufacturer, is not guaranteed or endorsed by the publisher.
